# Utilization of *Plantago major* seed mucilage containing *Citrus limon* essential oil as an edible coating to improve shelf‐life of buffalo meat under refrigeration conditions

**DOI:** 10.1002/fsn3.2137

**Published:** 2021-01-19

**Authors:** Mohammad Noshad, Behrooz Alizadeh Behbahani, Hossein Jooyandeh, Mostafa Rahmati‐Joneidabad, Mohsen Ebrahimi Hemmati Kaykha, Mitra Ghodsi Sheikhjan

**Affiliations:** ^1^ Department of Food Science and Technology Faculty of Animal Science and Food Technology Agricultural Sciences and Natural Resources University of Khuzestan Mollasani Iran; ^2^ Department of Horticultural Science Faculty of Agriculture, Agricultural Sciences and Natural Resources University of Khuzestan Mollasani Iran

**Keywords:** antibacterial activity, antioxidant effect, buffalo meat, edible coating, shelf‐life extension

## Abstract

This study is aimed to develop a novel edible coating based on *Plantago major* seed mucilage (PMSM) and *Citrus limon* essential oil (CLEO) to increase the shelf‐life of buffalo meat during cold storage. The CLEO was firstly isolated by the hydrodistillation method, and it contained mainly limonene (40.5%) and carene (15.4%) with remarkable antioxidant activity (55.7%, 63.8%, and 51.85% based on the DPPH‐radical scavenging, ABTS‐radical scavenging, and carotene‐linoleic acid bleaching tests, respectively) and antibacterial effect against some pathogenic and spoilage microorganisms. The CLEO (0%, 0.5%, 1%, 1.5%, and 2%) was then incorporated into PMSM solution to develop a novel CLEO‐loaded PMSM edible coating for improving the shelf‐life of buffalo meat. The edible coating was able to significantly reduce the progression of lipid oxidation (peroxide value) and microbial growth (total viable count, psychrotrophic bacteria, *Escherichia coli*, *Staphylococcus aureus*, and fungi) in buffalo meat during storage period of 10 days at 4°C in comparison with the control (noncoated sample). The meat hardness and sensory properties (i.e., odor, color, appearance, texture, and overall acceptability) were also maintained better upon edible coating applications. Based on the results, the CLEO‐rich PMSM edible coating could be applied as a new and effective preservative to improve the stability of meat products to lipid oxidation and microbial spoilage.

## Graphical abstract

Based on the results, the *Citrus limon* essential oil rich *Plantago major* seed mucilage edible coating could be applied as a new and effective preservative to improve the stability of meat products to lipid oxidation and microbial spoilage.

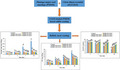

## 1 INTRODUCTION

Buffalo meat is considered as an alternative source of meat and a healthy choice due to the absence of hormones and stimulants. It is also rich in iron and protein and has lower contents of fat and cholesterol in comparison with skinless chicken, pork, and beef meats. It is also worth noting that buffalo meat has a red brilliant colour because of very low contents of intramuscular fat. However, buffalo meat, as other types of fresh meat, is a very perishable food. And the presence of spoilage and pathogenic microorganisms on the surface of buffalo meat could affect safety, hygienic quality, and shelf‐life of the product (Cannarsi et al., 2008). Furthermore, buffalo meat (raw and cooked) could undergo lipid oxidation and subsequent deterioration in colour, odor, and flavour quality and nutritional value (Descalzo et al., 2008; Juárez et al., 2010).

Modern packaging technologies have the potential to prolong the shelf‐life of food products through inhibiting or delaying lipid oxidation and microbial growth. Synthetic packaging have been extensively applied for these purposes; however, due to the negative impact of the residues of these packaging systems on the environment, food industry experts have been motivated to develop novel environmentally friendly packaging systems such as edible films and coatings to preserve food quality during storage (Heydari et al., 2020). Currently, the natural polymer‐originated edible coatings, particularly polysaccharide‐based ones, are used as biodegradable and environmentally friendly packaging to preserve the quality, minimize the loss of volatile nutraceuticals and moisture, and increase the storage stability of food products (Alizadeh Behbahani, Yazdi, Shahidi, Mortazavi, et al., 2017; Vital et al., 2018).

*Plantago major* is a flowering plant from the family *Plantaginaceae*. It has a large amount of small and ellipsoidal seeds, and the presence of polysaccharides in the seed coat make it to be gummy under warm and humid conditions. The polysaccharides are comprised of arabinose, xylose, rhamnose, galactose, glucose, glucuronic acid, and galacturonic acid and have the ability to be used as active natural polymers for developing edible coatings (Alizadeh Behbahani, Shahidi, et al., 2017). Currently, antimicrobial and antioxidant edible coatings are receiving much attention and application due to environmental issues. In this way, the edible coatings loaded with plant essential oils and extracts have been successfully developed for inhibiting or reducing oxidation and microbial growth in various food products (Choulitoudi et al., 2017; Grosso et al., 2020; Shin et al., 2017).

*Citrus limon* belongs to the Rutaceae family with yellow edible fruits and evergreen leaves. It is rich in vitamin C, and its juice is traditionally applied to treat fevers, sore throats, scurvy, chest pain, and high blood pressure (Klimek‐Szczykutowicz et al., 2020). The *C. limon* essential oil contains limonene (the main component of the essential oil), β‐pinene, γ‐terpinene, sabinene, linalool, myrcene, geranial, and so forth. The limonene‐loaded edible coatings have been used as antimicrobial and antioxidant agents to increase the shelf‐life different food products (Dhital et al., 2018; Maleki et al., 2018).

In recent years, global demands have been raised for marinated meat products. The reasons for this are majorly associated with the nutritional properties, the extended shelf‐life in addition to the enhancement of the sensory and textural properties of this type of food (Barbut et al., 2008; Siroli et al., 2020). Moreover, marination makes it possible to variegate meat products and to present more choices to consumers. Marination is a broadly employed technic in the meat industry comprising the injection or immersion of meat cuts into aqueous solutions consisting of a variety of components like water, salt, lemon juice, vinegar, brine, soy sauce, herbs, essential oils, tenderizers, spices, and organic acids (Garcia‐Marquez et al., 2012; Kargiotou et al., 2011; Siroli et al., 2020). In general, marination of a meat cut is normally carried out to enhance the production efficiency (i.e., by elevating the product moisture content), ameliorate the sensory properties of the resulting product, and, eventually, restrict (or at least postpone) oxidative reactions (Alvarado & McKee, 2007; Siroli et al., 2020; Vlakhova‐Vangelova & Dragoev, 2014; Yusop et al., 2010). Furthermore, earlier works have shown that the marinade solutions containing “natural” components (e.g., spices, herbs, essential oils) can have antimicrobial effect on pathogens and spoilage microorganisms in poultry, beef, and pork meat. Apart from their capability of enhancing the safety and shelf‐life of marinated meat, the application of ingredients like essential oils may also improve consumers’ willingness to purchase, in light of the recent growing attitude towards the consumption of clean‐label products (Asioli et al., 2017; Karam et al., 2019; Pathania et al., 2010; Siroli et al., 2020). For instance, it was revealed that citrus juice marinade (31% orange juice, 31% lemon juice, 38% distilled water) could give rise to the beef weight, sensory scores, and the solubilization of collagen (Burke & Monahan, 2003).

Consequently, in this research, the essential oil of lemon was utilized as a natural compound to ameliorate the oxidative and microbial stability in addition to sensory attributes of meat. Nevertheless, essential oils normally have strong flavor or odor, which restricts their direct usage in foodstuffs. In this regard, edible coatings could be applied for the encapsulation of essential oils and reduction of their negative impacts on sensory properties and consumer acceptance of food products (Barzegar et al., 2020). Recently, there has been a growing tendency towards the manufacture of natural polysaccharide‐based biodegradable and edible coatings. Plant seeds are the common and ancient sources of mucilage. *Plantago major* is one of the most frequent and vastly distributed pharmaceutical products all over the world, which can be found in many areas of the world; its seeds have been used from ancient times as an anti‐infective, immune‐modulating, anti‐inflammatory, analgesic, anti‐microbial, anti‐ulcerogenic, antioxidant, and anti‐cancer agent, as well as for wound treating purposes (Alizadeh Behbahani, Yazdi, Shahidi, Hesarinejad, et al., 2017).

To the best of our knowledge, there is no evidence about the role of the oxidative and microbial stability of buffalo meat wrapped by CLEO‐loaded PMSM edible coating. The present study is the carrier capability of the PMSM has been tested for lemon essential oil, to produce a simple, environmentally, and inexpensive bioactive edible coating for improving the microbial and oxidative stability, as well as the sensory characteristics of buffalo meat during cold storage.

## 2 MATERIALS AND METHODS

### 2.1 Materials

*Citrus limon* fruits and *P. major* seeds were purchased from Dezful city (Khuzestan, Iran) and a local market (Khuzestan, Iran), respectively. Linoleic acid, β‐carotene, DPPH (2,2‐diphenyl‐1‐picrylhydrazyl), ABTS (2,2′‐Azino‐bis (3‐ethylbenzothiazoline‐6‐sulfonic acid) diammonium salt), gallic acid, and quercetin were supplied from Sigma‐Aldrich Co. Mueller Hinton Agar (MHA), Mueller Hinton Broth (MHB), Sabouraud Dextrose Agar (SDA), Sabouraud Dextrose Broth (SDB), Eosine Methylene Blue (EMB), Plate Count Agar (PCA), and Mannitol Salt Agar (MSA) were acquired from Merck Co. All other chemical reagents and materials applied in the current study were of analytical grade.

### 2.2 Essential oil extraction and characterization

#### 2.2.1 Extraction

The peels of *C. limon* fruits were dried at room temperature and then powdered by means of a miller. After that, the peel powder (50 g) was transferred to a Clevenger apparatus containing 750 ml distilled water and the hydrodistillation‐based extraction process was completed (3 hr). The obtained *C. limon* essential oil (CLEO) was collected in glass vials and then stored at 4°C (Alizadeh Behbahani, Shahidi, et al., 2017).

#### 2.2.2 Gas chromatography‐mass spectroscopy (GC‐MS)

The CLEO was subjected to a gas chromatograph (GC; Agilent 7890A) coupled to a mass spectrometer (MS; Agilent 5975C) to identify its main chemical compounds, according to the following conditions: 0.2 µl injection volume, 5°C/min heating rate, 70 eV ionization energy, helium gas with 1.1 ml/min rate, and DB‐5 capillary column (30 m × 0.25 mm × 0.25 µm). The obtained retention profiles were finally compared with those of known samples analyzed by a GC‐MS apparatus with similar conditions (Alizadeh Behbahani et al., 2019a; Heydari et al., 2020).

#### 2.2.3 Fourier transform infrared spectroscopy

The functional groups of active compounds of the CLEO were analyzed by a Fourier transform infrared spectroscopy (FTIR) spectrometer. To do this, the CLEO was mixed with potassium bromide and compressed to obtain an appropriate pellet. The pellet was then subjected to the FTIR spectrometer, and the CLEO spectrum was collected from 400 to 4,000 wavenumber range (Alizadeh Behbahani, Falah, et al., 2020).

#### 2.2.4 Total phenolic contents (TPC)

The method of Ahmed et al. (2019) with some changes was employed to measure the TPC of CLEO. The oil (0.2 ml) or gallic acid (0.2 ml; 0–0.5 mg/ml) was mixed with 10% Folin‐Ciocalteu's reagent (2.5 ml). Then, Na_2_CO_3_ (2 ml; 7.50%) was added and the solution was incubated for 1 hr at ambient temperature. The absorbance was read at 756 nm, and the TPC of the CLEO was calculated and expressed as mg gallic acid equivalent (GAE) per g of CLEO.

#### 2.2.5 Total flavonoid contents

The total flavonoid contents (TFC) of the CLEO was measured based on the method of Saki et al. (2019). Briefly, the sample (0.5 ml) was charged with 300 µl of NaNO_2_ solution (1:20 w/v) and the mixture was vortexed for 10 s and stored at room temperature for 5 min. In the next step, AlCl_3_ (300 µl; 1:10 w/v), NaOH (1 M), and distilled water (1.9 ml) were added and mixed for 10 s. The absorbance of the mixture was read at 510 nm, and the TFC of the CLEO was expressed as mg quercetin equivalent (QE) per g of CLEO.

#### 2.2.6 Antioxidant activity

DPPH‐radical scavenging (DPPH‐RS) activity of the CLEO was investigated by utilizing the method of Wollinger et al. (2016). To do this, 0.05 ml of CLEO was mixed with 3.95 ml of methanolic DPPH solution (0.1 mg/ml = 250 µM) followed by incubation at room temperature and in a dark place for 60 min. The methanol (0.05 ml) was applied to prepare the blank sample in the same way. The absorbance of the sample (As) or blank (Ab) was measured at 518 nm, and the DPPH‐RS activity was calculated as below:

DPPH-RSactivity(%)=[1-As/Ab]×100

The method of Shan et al. (2005) with minor modification was used to determine the ABTS‐radical scavenging (ABTS‐RS) activity of the oil. Briefly, the same volumes of 7 mM ABTS solution and 2.45 mM K_2_S_2_O_8_ were mixed together and kept at 25°C for 16 hr under dark conditions. The obtained ABTS radical cation solution was then charged with methanol to reach 0.7 ± 0.2 absorbance at 734 nm. After that, the oil (0.1 ml) was mixed with the ABTS radical solution (3.9 ml) and the resulting solution was kept at ambient temperature for 6 min, which was followed by measuring its absorbance at 734 nm (As) against blank sample (methanol; Ac). The ABTS‐RS activity of the CLEO was calculated according to the following formula:

ABTS‐RSactivity(%)=[1‐As/Ac]×100

The inhibitory effect of the oil against bleaching of β‐carotene‐linoleate solution was evaluated according to the spectrophotometric method of Dapkevicius et al. (1998). The absorbance of the solution was determined at 490 nm after 120‐min incubation (As). The control sample was prepared in the same way, and its absorbance (Ac) was recorded at the time zero and after 120‐min reaction. The antioxidant potential of the CLEO was then measured as below:

$$\text{Inhibitory}\;\text{effect}\;\left(\%\right)=\left[\left({\text{As}}_{\left(120\right)}‐{\text{Ac}}_{\left(120\right)}\right)/\left({\text{Ac}}_{\left(0\right)}‐{\text{Ac}}_{\left(120\right)}\right)\right]\times 100$$

#### 2.2.7 Antibacterial activity

Antibacterial effect of the oil was evaluated against some pathogenic and spoilage bacteria (*Pseudomonas aeruginosa*, *Staphylococcus aureus*, *Staphylococcus epidermidis*, *Escherichia coli*, *Salmonella typhi*, *Listeria innocua*, *Bacillus subtilis*, and *Bacillus cereus*) *via* disk diffusion agar (DDA), well diffusion agar (WDA), minimum inhibitory concentration (MIC), and minimum bactericidal concentration (MBC) antimicrobial tests, according to the procedures reported by Noshad et al. (2018).

### 2.3 Mucilage extraction

The mucilage of *P. major* seeds (PMSM) was extracted based on the optimized method of Alizadeh Behbahani, Yazdi, Shahidi, Hesarinejad, et al. (2017), according to the following conditions: water to seed ratio of 60:1, pH 6.8, temperature 75°C, and 1.5‐hr extraction time. The PMSM was collected from the seed surfaces *via* an extractor, filtered, dried (45°C overnight), milled, sieved, and kept under cool and dry conditions.

### 2.4 Preparation of CLEO‐loaded PMSM edible coating

The PMSM (2 g) was mixed with Tween‐80 (1 ml) and made up to 100 ml by distilled water followed by stirring and heating. Afterward, the PMSM solution was charged with CLEO (0, 0.5, 1, 1.5, and 2% v/v). The buffalo meat slices were then immersed in the PMSM‐CLEO solutions for 1 min, air‐dried (10 min, 25°C), and stored at 4°C for 10 days. The coated samples were analyzed in terms of their physicochemical, textural, microbial, color, and sensory changes at time intervals of 0, 1, 4, 7, and 10 days.

### 2.5 Physicochemical changes of the coated meat

#### 2.5.1 Changes in pH value

The meat sample (10 g) was blended with distilled water (90 ml) and homogenized (13,000 rpm, 30 s). The pH changes of the meat samples were then monitored by a pH meter (Dragon Lab, MX‐S) at room temperature (Heydari et al., 2020).

#### 2.5.2 Changes in moisture content

The moisture content of the meat samples was measured by the oven drying method (AOAC, 1995).

#### 2.5.3 Changes in peroxide value

The method of Alizadeh Behbahani and Imani Fooladi (2018a) was utilized to monitor the changes in peroxide value of samples during storage period.

### 2.6 Microbiological analysis of coated beef

The meat sample (5 g) was mixed with 0.1% peptone water (45 g) in a Stomacher and homogenized (200 rpm, 1.0 min). The subsequent dilutions (10^–1^ to 10^–6^) were then prepared in the test tubes containing 0.1% peptone water. The dilutions were inoculated into the plates containing culture medium and to perform the following microbial tests: total viable count (TVC) bacterial count in PCA (48 hr incubation at 37°C), psychrotrophic count (PTC) in PCA (10 days of incubation at 7°C), *S*. *aureus* count in MSA (24‐hr incubation at 37°C), *E. coli* count in EMB (24‐hr incubation at 37°C), and Fungi count in SDA (72‐hr incubation at 27°C) (Alizadeh Behbahani & Imani Fooladi, 2018b).

### 2.7 Textural changes

A Stable Micro System Texture Analyzer (TA, XT2i, UK) was applied to determine the hardness changes of the meat samples during cold storage. To do this, the samples (2 × 2 × 2 cm) were compressed by a probe (36 mm in diameter) up to 50% of their initial heights at 5 mm/s test speed, and the highest force (N) was considered as the tissue hardness of the samples (Heydari et al., 2020).

### 2.8 Color changes

The color of the meat samples coated by CLEO‐loaded PMSM was measured by means of a digital Chroma meter (Konica Minolta, CR‐400, Japan). The device was firstly calibrated by a white tile standard, and the color indices (L*, b*, and a*) and total color difference (Δ*E*) of the samples were then measured by the instrument (Realini et al., 2017):

$$\Delta E=\sqrt{{\left(\Delta L*\right)}^{2}+{\left(\Delta b*\right)}^{2}+{\left(\Delta a*\right)}^{2}}$$

### 2.9 Sensory evaluation

The samples were coded randomly with 3‐digit numbers, and then, their color, odor, appearance, texture, and overall acceptance were assessed by 20 semi‐trained panelists according to a nine‐point hedonic scale test (1 = dislike extremely to 9 = like extremely). The sensory score higher than 4 was considered as acceptable (Heydari et al., 2020).

### 2.10 Statistical analysis

The experiments were repeated three times. The data were analyzed by SPSS software (version 26), and Duncan test at 95% confidence level was applied to determine differences between data means.

## 3 RESULTS AND DISCUSSION

### 3.1 CLEO characterization

#### 3.1.1 Chemical composition

A total of 19 compounds were identified in the CLEO by the GC‐MS technique, which constituted 93.26% of the total oil (Table 1). Limonene (40.5%) was the main compound of the CLEO, followed by carene (15.4%), α‐terpineol (7.61%), terpinen‐4‐ol (6.16%), β‐bisabolene (5.53%), octadecenoic acid (3.55%), and caryophyllene (3.11%). Similarly, Jomaa et al. (2012) reported that limonene (61.8%–73.8%) is the main identified compound in the CLEO extracted from the fruit peel, and γ‐terpinene (9.4%–10.4%), β‐pinene (3.7%–6.9%), O‐cymene (1%–2.4%), and citral (0.8%–5.4%) are found at lower concentrations in the oil. Also, it was demonstrated that the main components of the CLEO’s fruit peel are monoterpenoids, such as limonene (69.9%), pinene (11.2%), terpinene (8.21%), sabinene (3.9%), myrcene (3.1%), geranial (E‐citral, 2.9%), neral (Z‐citral, 1.5%), and linalool (1.41%) (Klimek‐Szczykutowicz et al., 2020). Although limonene has been found as the major compound of CLEO in all above‐mentioned studies, the minor differences between the concentrations of the chemical compounds could be due to fact that the quality and quantity of the essential oil is mainly dependent on the variety, growth stage, climate, growth location, and collection time of the bioactive plant (Kiarsi et al., 2020).

**TABLE 1:** Chemical compositions of *Citrus limon* peel essential oil (CLEO)

No.	Compounds	Retention time (min)	%
1	Limonene	3.42	40.5
2	Carene	3.58	15.4
3	Bicyclo [2.2.1] heptan	4.10	0.75
4	Endo‐Borneol	4.59	0.42
5	Terpinen‐4‐ol	4.68	6.16
6	α‐Terpineol	4.80	7.61
7	Carvone	5.29	0.8
8	Geranyl acetate	6.24	0.87
9	Caryophyllene	6.88	3.11
10	Farnesene	7.04	0.76
11	Humulene	7.16	1.4
12	β‐Copaene	7.38	0.99
13	α‐Farnesene	7.48	2.62
14	β‐Bisabolene	7.54	5.53
15	Alloaromadendrene	8.03	0.46
16	Caryophyllene oxide	8.26	0.79
17	Hexadecanoic acid	10.53	0.91
18	Octadecenoic acid	11.68	3.55
19	Methyl stearate	11.82	0.63
Total			93.26

#### 3.1.2 Structural analysis

**FIGURE 1:**
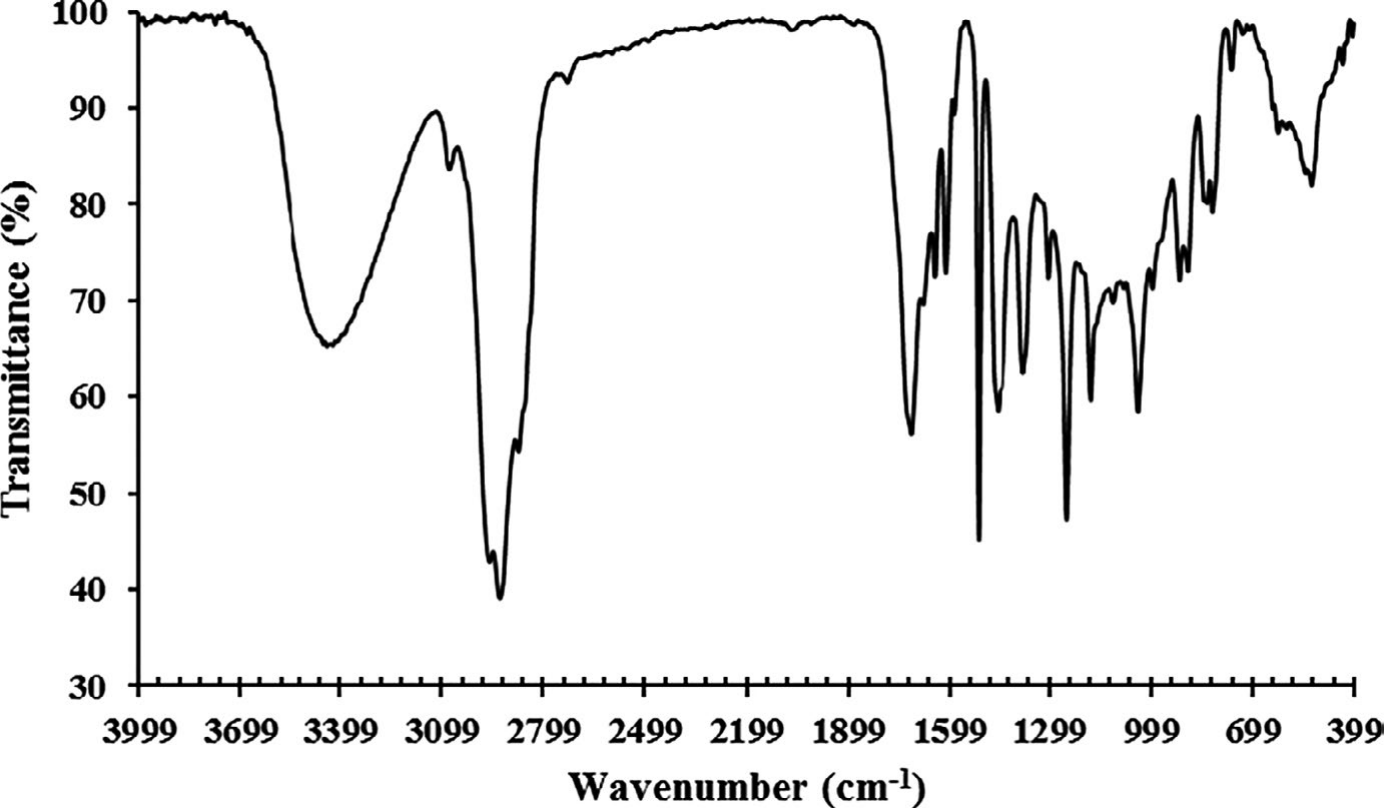
FTIR spectrum of *Citrus limon* essential oil (CLEO)

The CLEO was subjected to FTIR spectroscopy for identifying its functional groups (Figure 1). The broad peak around 3,437 cm^−1^ is likely due to –OH stretching, the strong peak at 2,927 cm^−1^ is due to –CH stretching, and small peaks located at 1,377, 1,454, 1,642 cm^−1^ could be assigned to the C=C aromatic ring bending of the bioactive compounds of the CLEO (Sotelo‐Boyás et al., 2017). Additionally, it was claimed that the characteristic peak indicating the bending vibration of =CH for the disubstituted double bond of the limonene is located around 889 cm^−1^ (Akolade et al., 2020). It is also noteworthy that the peaks at 3,076 cm^−1^ and 2,872–2,962 cm^−1^ could be probably attributed to the = C‐H and aliphatic C‐H of the limonene (Ansarifar et al., 2019). Likewise, some peaks were observed at 1,247 cm^−1^ (=C‐H in‐plane bending of aromatic rings and ‐CH_2_ swing in alkanes), 1,176 cm^−1^ (C‐O stretching vibration), 1,036 cm^−1^ (C‐OH deformation vibration), and 759 cm^−1^ (=CH vibration of benzene ring) (Alizadeh Behbahani et al., 2019b). These aforementioned characteristic peaks indicate the presence of phenolic and aromatic compounds in the CLEO.

#### 3.1.3 TPC and TFC

The mean values of TPC and TFC of CLEO were 38.91 ± 0.44 mg GAE/g and 87.90 ± 0.63 mg QE/g, respectively. According to the results of Abad‐García et al. (2012), five different classes of phenolic compounds are found in *Citrus* species: flavanone (such as O‐dihexoside of naringenin), flavones (such as luteolin‐6‐C‐glucoside, apigenin‐7‐O‐rutinoside‐4′‐O‐glucoside, apigenin‐6‐C‐hexoside‐O‐hexoside, luteolin‐7‐O‐neohesperidoside‐4′‐O‐glucoside, and aligenin‐8‐C‐hexoside‐O‐acylrhamnoside), flavonols (such as 7‐O‐rutinosides of isorhamnetin, kaempferol, quercetin, and tamarixetin, isorhamnetin‐3‐O‐rutinoside‐7‐O‐glucoside, kaempferol‐3‐O‐rutinoside, and tamarixetin‐3‐O‐rutinoside‐7‐O‐glucoside), hydroxycinnamic acids (such as O‐hexoside of sinapic and ferulic acids), and coumarins (such as O‐rhamnosylhexoside and O‐hexoside of scopoletin). The high contents of phenolic compounds have been found in *citrus* species essential oils and extracts (Di Rauso Simeone et al., 2020; Hojjati & Barzegar, 2017; Sultana et al., 2015). The phenolic compounds act as electron donors in free radical reactions and are often correlated to the antioxidant effect of the essential oils.

#### 3.1.4 Antioxidant activity

It has been demonstrated that a synergy among chemical compounds of plant essential oils determines their antioxidant potential and this bioactive activity is primarily influenced by the main compounds of the oil (Alizadeh Behbahani, Noshad, et al., 2020; Farahmandfar & Tirgarian, 2020). The CLEO was therefore subjected to different in‐vitro antioxidant assays, namely DPPH‐RS activity, ABTS‐RS activity, and β‐carotene bleaching, to evaluate its potential antioxidant activity. In DPPH‐RS activity method, potent antioxidants react with DPPH free radicals and convert them to nonradical DPPH‐H molecules and the intense violet color of the reaction medium is subsequently changed to the colorless one (Fan et al., 2012). The CLEO had a remarkable DPPH‐RS activity (55.70 ± 0.46%), representing the potential of the bioactive CLEO to neutralize free radicals, mainly due to electron donation. The ABTS‐RS activity is however based on the reduction of ABTS^●+^ to ABTS, through hydrogen atom transfer mechanism, in the presence of antioxidant agents (Jordão et al., 2012). The ABTS‐RS activity of the CLEO was found to be 63.82 ± 0.89%. This means that the CLEO is able to scavenge ABTS free radicals *via* transferring hydrogen atoms. The inhibitory effect of the oil against β‐carotene discoloration was remarkable, as well (51.85 ± 0.67%). Potential antioxidant activity of the CLEO is therefore due to the presence of phenolic compounds and limonene in the oil as their antioxidative function (*via* electron and hydrogen atom donation) have been reported in the literature (Baschieri et al., 2017; Campêlo et al., 2011; Roberto et al., 2010). Accordingly, the CLEO could be employed as a natural antioxidant to inhibit lipid oxidation reactions and improve oxidative stability of food products.

#### 3.1.5 Antimicrobial activity

**FIGURE 2:**
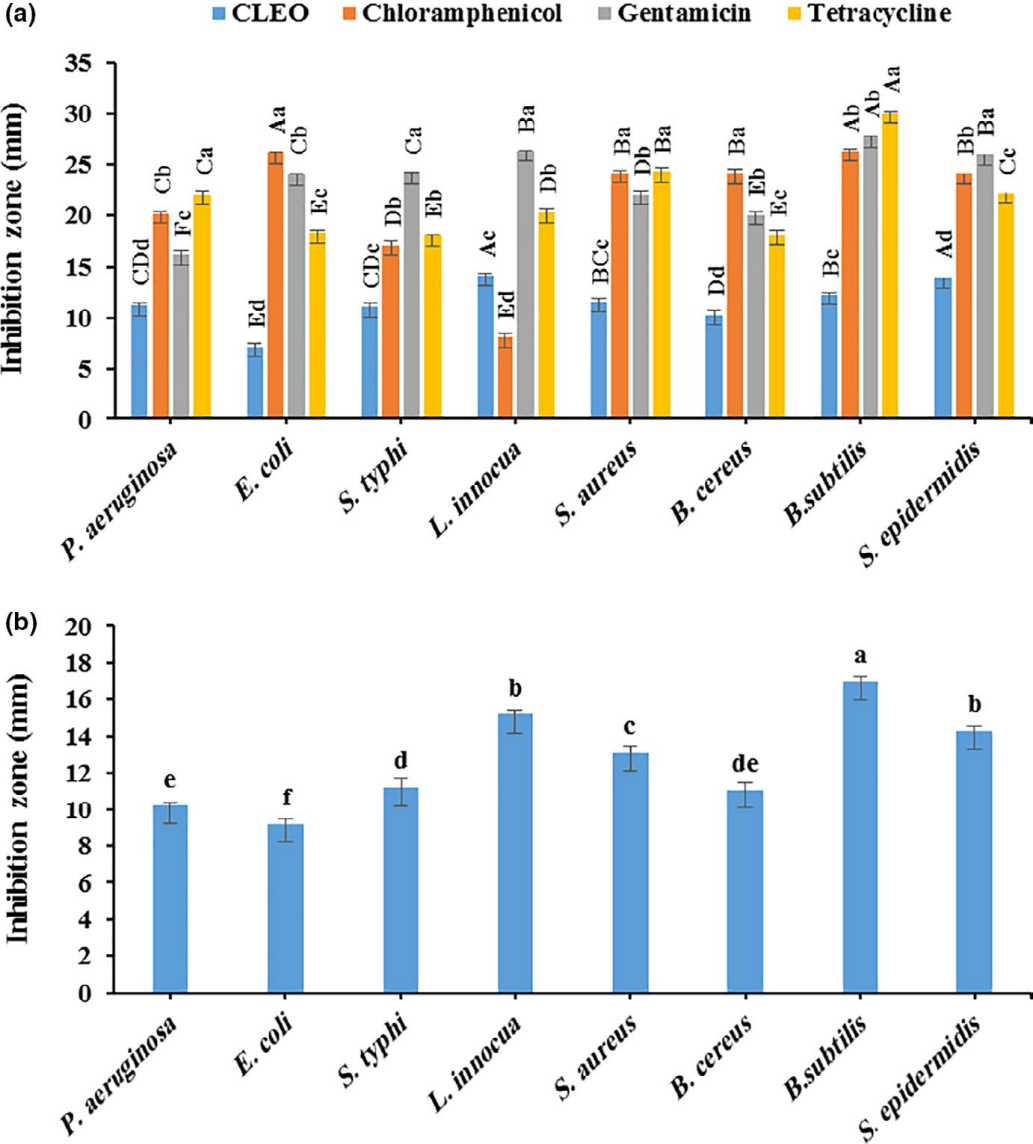
Antimicrobial activity of *Citrus limon* essential oil (CLEO) against some pathogenic and spoilage bacteria species, according to the disk diffusion agar (DDA) test (a) and well diffusion agar (WDA) test (b)

The antimicrobial effect of the CLEO on the spoilage and pathogenic microorganisms is illustrated in Figure 2. The oil had a remarkable antibacterial effect against Gram‐positive and Gram‐negative bacterial species; however, its effect was dependent on the microorganism type. In the DDA test, *L. innocua* and *S. epidermidis*, with the inhibition zones (IZ) of 14.10 and 13.90 mm, respectively, were the most sensitive species to the CLEO; whilst, the most resistant microorganism was *E. coli* with IZ of 7.20 mm (Figure 2a). It could be also noted that the Gram‐positive bacterial species were generally more sensitive than the Gram‐negative ones (12.44 mm vs. 9.83 mm IZ). In general, the CLEO had significantly lower inhibitory effect (IZ = 11.46 mm) compared to the common antibiotics gentamicin (IZ = 23.37 mm), tetracycline (IZ = 21.66 mm), and chloramphenicol (IZ = 20.15). According to the results of WDA antimicrobial assay, *B. subtilis* and *E. coli* were the most sensitive and resistant species to the CLEO, respectively (Figure 2b). And the average inhibition zone was found to be 10.2 mm for Gram‐negative bacteria and 14.4 mm for Gram‐positive bacteria. This difference in the sensitivity of the microorganisms to the CLEO could be mainly attributed to the complex structure of lipopolysaccharide cell membrane of the Gram‐negative bacteria compared to the single‐layer mucopeptidic structure of the Gram‐positive's cell membranes, which have the potential to decrease the diffusion of hydrophobic compounds across the membrane (Alizadeh Behbahani & Imani Fooladi, 2018a; Alizadeh Behbahani et al., 2018; Yeganegi et al., 2018).

The results of MIC and MBC tests are indicated in Table 2. In agreement with the DDA and WDA findings, the growth of *L. innocua* was suppressed to a greater extent than the other microorganism. The antibacterial effect of the CLEO could be due to the presence of limonene (as the main chemical constituent) and phenolic compounds in the oil, which their antibacterial activity has been reported in some studies (Dholwani et al., 2008; Han et al., 2020; Liu et al., 2020).

**TABLE 2:** The minimum inhibitory concentration (MIC) and minimum bactericidal concentration (MBC) of the *Citrus limon* essential oil (CLEO) on some pathogenic microorganisms

Microorganism	MIC (mg/ml)	MBC (mg/ml)
*Pseudomonas aeruginosa*	400	>400
*Escherichia coli*	400	>400
*Salmonella typhi*	>400	>400
*Listeria innocua*	200	>400
*Staphylococcus aureus*	400	>400
*Bacillus cereus*	400	>400
*Bacillus subtilis*	400	>400
*Staphylococcus epidermidis*	400	>400

### 3.2 Physicochemical changes of the coated buffalo meat

#### 3.2.1 pH changes

**FIGURE 3:**
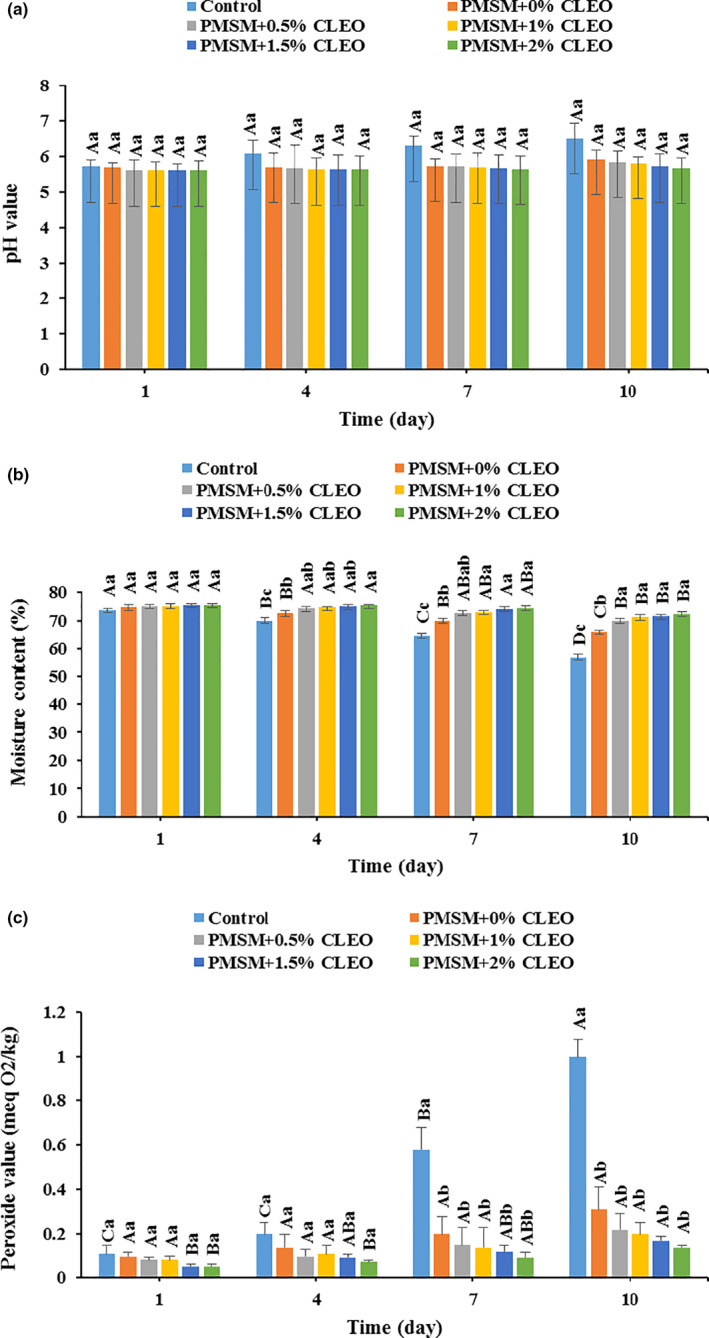
Changes in pH (a), moisture content (b), and peroxide value (c) of the buffalo meat during cold storage

Figure 3a shows the pH change of the coated buffalo meats during storage period at 4°C. Despite the fact that the samples underwent a gradual pH increase during storage, there were no significant differences among the samples (*p* > .05). The initial pH value of the control sample (noncoated buffalo meat) was 5.7 and it reached 6.5 by the end of storage time (*p* > .05). The application of edible coating on the meat samples resulted in lower pH changes, and the CLEO‐loaded coatings, especially 2% CLEO loaded ones (PMSM + 2%CLEO) were more efficient than the others; the buffalo meat coated by PMSM + 2%CLEO showed the lowest changes in pH value (5.6–5.66) as the storage period rose from 1 to 10 days, compared to the control sample (*p* > .05). Indeed, microbial‐based enzymatic activities have the potential to degrade meat proteins to nitrogenous compounds like trimethylamine and ammonia with basic nature and the pH value could be subsequently increased during storage. However, the edible coatings rich in essential oils could reduce the permeability of carbon dioxide from the coating and the accumulation of this acidic compound could therefore lead to pH decrease in the sample and in turn microbial growth suppression (Alizadeh Behbahani, Noshad, et al., 2020). These findings are supported by the results of other studies (Barzegar et al., 2020; Heydari et al., 2020; Kiarsi et al., 2020).

#### 3.2.2 Moisture content changes

As can be seen from Figure 2b, all samples underwent a significant decrease in moisture content as a function of storage time (*p* < .05). The control sample showed an approximately 22.95% water loss at 10 days of storage; however, the water loss in the coated samples ranged from 11.43% in PMSM + 0%CLEO to 4.33% in PMSM + 2%CLEO by the end of storage period. And the higher CLEO loads in the edible coating, the lower was the water loss. This means that the CLEO‐loaded PMSM coatings inhibited the weight loss of the buffalo meat efficiently and could be applied to preserve its freshness for a longer period of time. This positive effect of the edible coating could be due to its lower permeability to water vapor and physical barrier function (Xiong et al., 2020).

#### 3.2.3 Peroxide value changes

The changes of peroxide value of buffalo meat coated by the CLEO‐PMSM are illustrated in Figure 3c. The control and coated meat samples experienced an increase in the peroxide value as the storage time increased. By the end of storage period, there were significant differences in the peroxide value between the control meat and the wrapped ones, representing lower peroxide values of 0.31, 0.22, 0.2, 0.17, and 0.14 meq O_2_/kg in PMSM + 0%CLEO, PMSM + 0.5%CLEO, PMSM + 1%CLEO, PMSM + 1.5%CLEO, and PMSM + 2%CLEO, respectively, compared to the peroxide value of 1 meq O_2_/kg in the noncoated sample. It could be therefore concluded that the edible coating, especially high‐essential oil‐loaded ones, is effective in preventing the formation of hydroperoxide in the buffalo meat during storage period. This could be mainly due to the radical scavenging activity and hydroperoxide formation‐suppressing potential of essential oils (Barzegar et al., 2020). The inhibitory effects of the edible coatings rich in essential oil towards the formation of primary lipid oxidation products in meat products were reported in the literature (Alizadeh Behbahani, Noshad, et al., 2020; Wang et al., 2020).

### 3.3 Microbial load changes

**FIGURE 4:**
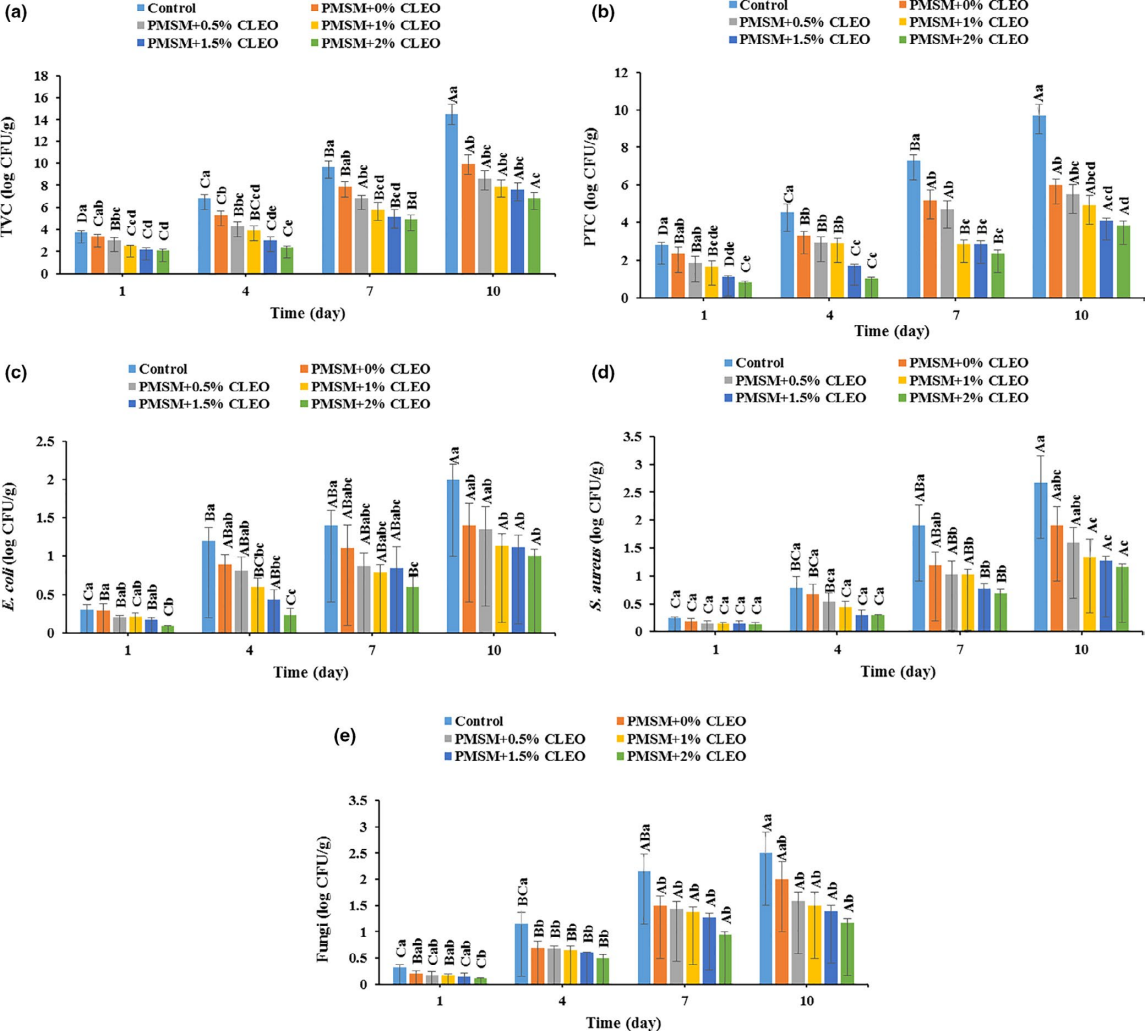
Changes in total viable count (a), psychrotrophic count (b), *Escherichia coli* count (c), *Staphylococcus aureus* count (d), and fungi count (e) of the buffalo meat stored at 4°C for 10 days

The effect of the PMSM coatings loaded with different CLEO concentrations on the TVC changes in buffalo meat during storage period is indicated in Figure 4a. The TVC increased significantly in all samples as the storage time increased (*p* < .05). The control sample had the highest TVC increment (~ 3.89‐fold) during storage, and the buffalo meat coated by PMSM + 2%CLEO presented the lowest change in TVC (~ 3.28‐fold); the higher the CLEO concentrations in the edible coatings, the lower were the changes in TVC of the meat samples. It is reported that 10^7^ CFU/g (7 log CFU/g) is the maximum recommendation limit of TVC for fresh meat (ICMSF, 1986). In this context, the sample wrapped with 2% CLEO‐loaded PMSM had a TVC of 6.83 log CFU/g by the day 10 of storage, whilst the TVC in the control sample exceeded the maximum recommendation limit at 7 days (9.62 log CFU/g). This indicates microbial shelf‐lives of about 4–6 days for the noncoated buffalo meat and more than 10 days for the 2% CLEO‐loaded PMSM‐coated meat. Thus, the CLEO‐rich edible coatings have the potential to increase the shelf‐life of buffalo meat significantly, mainly due to the antimicrobial activity of the oil, in line with other studies (Guerrero et al., 2020; Umaraw et al., 2020).

The PTC in buffalo meat was significantly influenced (*p* < .05) by the edible coating and storage time (Figure 4b). All the samples underwent a significant increase in the PTC during storage, and the highest (~ 6.89 log CFU/g) and lowest (~ 2.99 log CFU/g) PTC changes were observed in the control and PMSM + 2%CLEO coated meats, respectively. Indeed, the PTC indicated the same pattern as that of TVC, with control (noncoated) meat also being the highest by the storage time (9.70 ± 0.63 log CFU/g), followed by the buffalo meats coated by PMSM + 0%CLEO (6.00 ± 0.29 log CFU/g), PMSM + 0.5%CLEO (5.50 ± 0.5 log CFU/g), PMSM + 1%CLEO (4.93 ± 0.49 log CFU/g), PMSM + 1.5%CLEO (4.09 ± 0.17 log CFU/g), and PMSM + 2%CLEO (3.83 ± 0.26 log CFU/g). This positive effect of the CLEO‐loaded edible coating could be ascribed to the antimicrobial property of the CLEO and the oxygen barrier function of the coating. In this way, the growth of aerobic psychrotrophic bacteria (like *Pseudomonas* species), as the main cause of the meat spoilage under aerobic conditions, is suppressed under low‐oxygen pressure (Alghooneh et al., 2015; Barzegar et al., 2020).

The changes in *E. coli* and *S. aureus* populations of buffalo meat, including control and samples coated by PMSM and CLEO‐loaded PMSM, stored at 4°C for up to 10 days are indicated in Figure 4c,d. The initial *E. coli* and *S. aureus* counts in all samples were in the range of 0.09–0.30 and 0.13–0.24 log CFU/g, respectively. All the coated samples remarkably decreased the population of *E. coli* and *S. aureus* as compared with the control at the end of storage time; the maximum count of *E. coli* (2.00 log CFU/g) and *S. aureus* (2.67 log CFU/) were found in the noncoated buffalo meat, and the sample wrapped with PMSM + 2% CLEO showed the lowest *E. coli* (1 log CFU/g) and *S. aureus* (1.17 log CFU/g) populations (*p* < .05).

A similar behavior was observed for the growth pattern of fungi (Figure 4e). The CLEO‐rich PMSM coatings manifestly reduced the growth of fungi on the buffalo meat surface. The control and the sample coated by PMSM + 2% CLEO had the highest and lowest fungi populations of 2.50 and 1.17 log CFU/g at the end of storage period, respectively. This is mainly due to the fact that the oxygen‐barrier function of the edible coating makes it to be an inappropriate place for the growth of aerobic fungi species (Heydari et al., 2020). The anti‐fungal effect of the CLEO could be an another factor in reducing the population of fungi in buffalo meat during storage (Ammad et al., 2018). In accordance with our results, it has been reported that *Zataria multiflora* essential oil and grape seed extract reduced the growth of mesophilic, psychrotrophic, *Pseudomonas* spp., lactic acid bacteria, and yeast strains in raw buffalo patty during cold storage (Tajik et al., 2015).

### 3.4 Hardness changes

**FIGURE 5:**
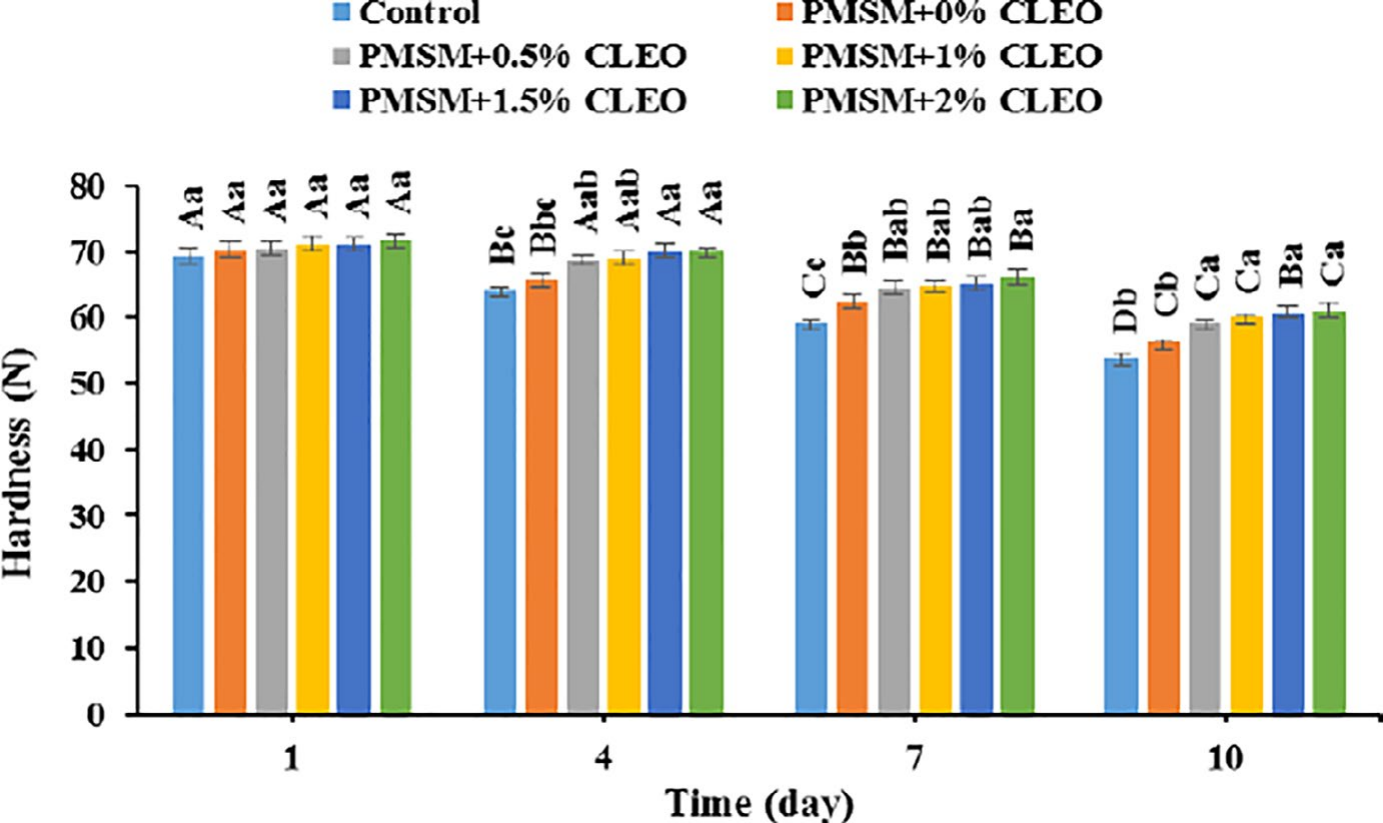
Changes in hardness of buffalo meat samples during 10 days storage at 4°C

The hardness of control and coated meat samples showed a decreasing trend throughout the storage period, and the hardness of the buffalo meat was not adversely influenced by the edible coating (Figure 5). The samples coated by PMSM, especially CLEO‐rich coatings, showed higher hardness compared with the noncoated one; the highest and lowest hardness losses were observed in the control and PMSM + 2% CLEO samples (22.40% vs. 14.83%). Indeed, the higher essential oil concentration in the edible coating, the higher was buffalo meat hardness. According to the study of Ghani et al. (2018), essential oils have the potential to inhibit the growth of microorganisms and the activity of endogenous enzymes of meat (e.g., collagenase, cathepsins, and calpains). Myofibrillar and collagen proteins could be therefore inhibited from the enzymatic degradation, so meat texture preserves over time. Similar results have been reported by Kiarsi et al. (2020) and Alizadeh Behbahani, Noshad, et al. (2020).

### 3.5 Color changes

**FIGURE 6:**
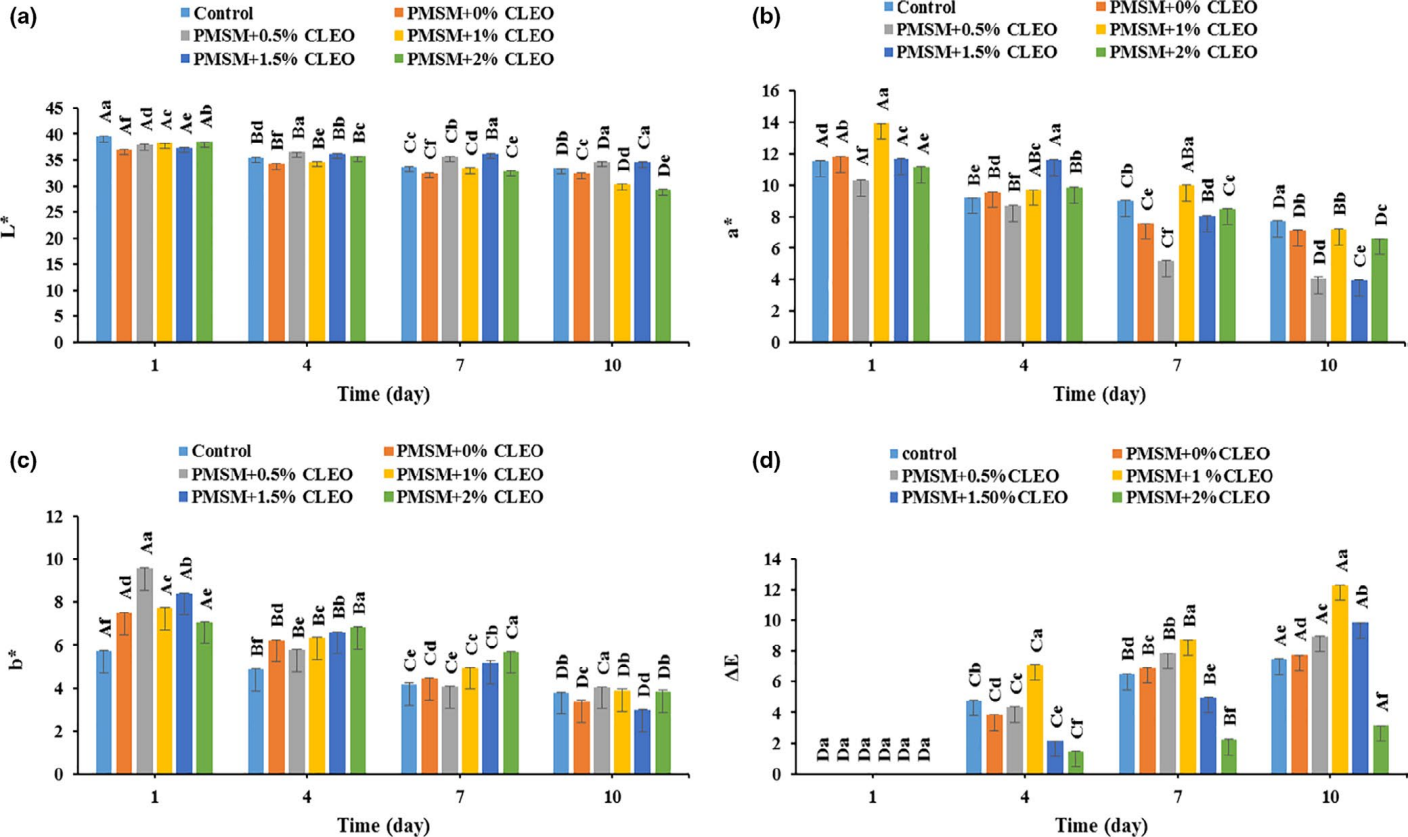
Changes in L* (a), a* (b), b* (c), and Δ*E* (d) of buffalo meat samples during 10 days storage at 4°C

Color is considered as one of the main factors in determining meat quality. L*, a*, b*, and ΔE values of the samples are provided in Figure 6a–d. As storage time increased from 1 to 10 days, there was a significant decrease in L* values, representing that the buffalo meat samples become darker (Figure 6a). However, the samples wrapped with PMSM + 0.5%CLEO and PMSM + 1.5%CLEO were significantly lighter at the end of storage period, compared with control and other coated pairs (*p* < .05). A significant decrease in a* value was also observed in all samples as a function of storage time and the samples become less red (Figure 6b). This was more pronounced in the coated samples, which could be related to the conversion of myoglobin to metmyoglobin under low‐oxygen pressure conditions of the PMSM coatings in conjugation with exudate accumulation in the coated samples (Vital et al., 2016).

It is also noteworthy that although the coated samples had initially higher b* values than the noncoated control one (likely due to the yellowish color of the coating), there was a significant decrease in b* values of the buffalo meat samples during storage (Figure 6c). This could be probably attributed to the fact that the samples became darker over time. Moreover, all of the meat samples underwent a significant increase in total color change as storage time increased (Figure 6d). And the PMSM + 2%CLEO‐coated buffalo meat had a smaller total change in comparison to the other counterparts, probably indicating its shelf‐life extension. In congruent with our study, it has been reported that coated meat samples that show a smaller total change would have an extension in shelf‐life (Antoniewski et al., 2007).

### 3.6 Sensory properties

**FIGURE 7:**
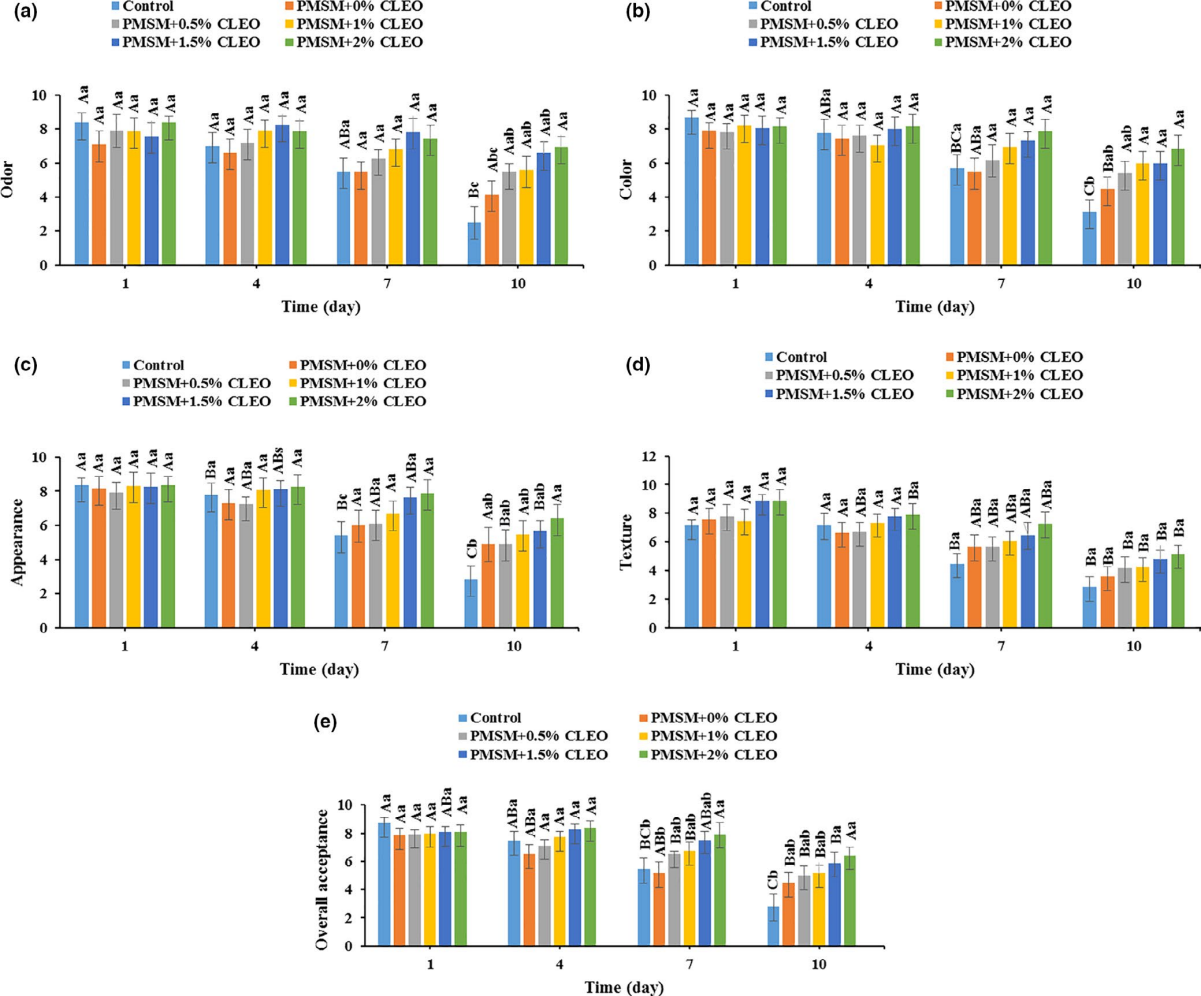
Changes in odor (a), color (b), appearance (c), texture (d), and overall acceptance (e) of buffalo meat samples during 10 days storage at 4°C

The CLEO‐loaded edible coatings did not affect the sensory properties of the buffalo meat over storage period (Figure 7a–e). From the panelist’ points of view, the meat samples with a sensory score above 4 could be accepted (Heydari et al., 2020). All of the sensory properties were decreased as storage time increased and the control sample was unacceptable after 10 days storage at 4°C. However, the coated buffalo meats were acceptable throughout the storage period, except for the PMSM coated sample, which it perceived a texture score of 3.58 at the end of storage time (Figure 7d). In general, the noncoated and coated meat samples had the shelf lives of 7 and 10 days, respectively, according to the overall acceptance results (Figure 7e). And the lowest and highest sensory scores were observed in the control and meat samples wrapped with PMSM + 2% CLEO, respectively. This could be confirmed by the lipid oxidation and microbial growth progressions in the samples; the PMSM + 2% CLEO‐coated meat sample had the highest stability to the lipid oxidation and microbial spoilage in comparison to the control sample. Indeed, the antioxidant and antimicrobial function of the essential oil‐loaded edible coatings make them to be used as food‐grade preservatives to inhibit the lipid oxidation and microbial growth of the meat and meat products, thereby improving their shelf‐lives (Guerrero et al., 2020; Raeisi et al., 2016).

## 4 CONCLUSIONS

The quality and shelf‐life of meat and meat products could be significantly affected by microbial growth and lipid oxidation. The edible coating based on *Plantago major* seed mucilage and *Citrus limon* essential oil effectively decreased the lipid oxidation, hardness loss, and microbial growth in the buffalo meat during cold storage. The coated meat had also a higher consumer acceptance compared to the control sample. Therefore, *Citrus limon* essential oil‐rich *Plantago major* seed mucilage based edible coatings have the potential to maintain/improve the characteristics of meat products during the shelf‐life.

## CONFLICT OF INTEREST

The authors have declared no conflict of interest.

## ETHICAL APPROVAL

This article does not contain any studies with human or animal subjects.

## Data Availability

The data that support the findings of this study are available from the corresponding author upon reasonable request.
